# ‘Professional-supportive’ versus ‘economic-operational’ management: the relationship between leadership style and hospital physicians’ organisational climate

**DOI:** 10.1186/s12913-021-06760-2

**Published:** 2021-08-17

**Authors:** Pål E. Martinussen, Tonje Davidsen

**Affiliations:** Department of Sociology and Political Science, Norwegian University of Science and Technology (NTNU), Trondheim, Norway

## Abstract

**Background:**

Health systems across the world have implemented reforms that call for a reconsideration of the role of management in hospitals, which is increasingly seen as important for performance. These reorganisation efforts of the hospitals have challenged and supplemented traditional profession-based management with more complex systems of management inspired by the business sector. Whereas there is emerging evidence on how medical professionals in their role as leaders and managers adapt to the new institutional logics of the health care sector with increasing demands for efficiency and budgetary discipline, no previous studies have investigated whether leaders’ emphasis on clinical or financial priorities is related to how hospital physicians’ view their working situation. The purpose of this study was therefore to examine the relationship between leadership style and hospital physicians’ organisational climate.

**Methods:**

We utilised data from a survey among 3000 Norwegian hospital physicians from 2016. The analysis used three additive indexes as dependent variables to reflect various aspects of the organisational climate: social climate, innovation climate and engagement at the workplace. The variables reflecting leadership style were based on an item in the survey asking the respondents to rate the leadership qualities of their proximate leaders (department chair) on 11 specific dimensions. We used factor analysis to identify two types of leadership styles: a traditional profession-based leadership style that emphasises the promotion of professional standards and quality in patient treatment, and a leadership style that reflects the emerging management philosophy with focus on economic administration and budgetary control. Controlling for demographic background, leader role, foreign medical exam and specialty, the empirical model was estimated via multivariate regression.

**Results:**

The results documented a clear relationship between leadership style and organisational climate: a ‘professional-supportive’ leadership style is associated with better social climate, innovation climate and engagement at the workplace, while an ‘economic-operational’ leadership style is associated with a poorer social climate.

**Conclusions:**

The cross-sectional study design makes it impossible to draw inferences about direction of causality and causal pathways. However, the positive relationship between professional-supportive leadership and organisational climate is a matter, which should be seriously considered regardless of direction of causality.

## Background

The concept of culture has a long tradition in organisational studies, and refers to the shared values, beliefs or perceptions held by the employees of an organisation [1]. The organisational culture of a hospital is important for obvious reasons: it affects hospital performance. The organisational culture reflects the employees’ commitment to the organisation, and thereby their willingness and motivation to put in the extra effort in their work tasks. Dedicated and motivated employees are likely to improve the performance of a hospital, while the lack of such may impact negatively on patient care [2]. The importance of culture in healthcare organisations is well documented: burnout and low job satisfaction is related to treatment quality, turnover and patient satisfaction [3–11]. Whereas leadership is seen as an important factor determining organisational culture, there is still a lack of studies investigating the effect of leadership in hospitals that focuses on the medical profession. In the wake of the recent market-inspired health reforms in many countries, both managers and hospital physicians have had to adapt to the new institutional logics of the healthcare sector, with increasing demands for efficiency and budgetary discipline [12–14]. The purpose of this paper was to investigate the role of leadership style for hospital physicians’ perceptions of organisational culture in the context of a recent market-inspired health reform.

Researchers on organisations have long sought to establish the importance of organisational structure and leadership for aspects such as profitability, efficiency, performance and organisations’ growth and survival. Particular attention has been paid to hospitals, in an attempt to link organisational characteristics to important objectives for patients and employees [15]. While it may be difficult to establish a clear causal relationship between leadership and macro indicators such as for instance economic performance or medical quality, leadership may still make an important difference at the individual level [16, 17]. Previous studies have therefore investigated the role of various aspects of leadership, such as support, encouragement, respect, acknowledgement, collaboration, motivation and innovation [17–24]. Unsurprisingly, the research shows that such leadership skills are favourable for quality, organisational commitment, job satisfaction, burnout and stress.

Much of the work has been dominated by research on the concept of transformational leadership introduced by Bass and Avolio [25] and has mainly focused on nursing leadership and nursing staff. Transformational leadership is defined as a leadership approach that focuses on effective leaders as the ones that transform the basic attitudes, values and beliefs of their employees to increase their performance [26]. Previous studies have documented that such management characteristics and leadership skills improve job satisfaction, sustain commitment to the organisation, and enhance retention in nurses, and that particularly when midlevel managers exhibit transformational leadership styles nurses tend to experience higher job satisfaction and be less prone to turnover [27–43]. A 2007 review of the relationship between nursing leadership and patient outcomes by Wong and Cummings [44] reported that positive leadership behaviours (transformational, empowering, supportive, etc.) were associated with increased patient satisfaction [45–47], reduced patient mortality [48, 49], reduced adverse events [48, 50, 51], and reduced complications [48, 51]. A later review of the literature from 2017, building mainly on data on nurses, managers and patients, also identified the significance of leadership styles and practices on patient outcomes, health care workforce and organisational culture, with leadership styles that reflected a collaborative, multifaceted and dynamic process (e.g., transformational, employee-oriented leadership) being more effective and promoting positive outcomes [52]. The most recent systematic review on leadership styles and nurses’ job satisfaction from 2021 confirmed this picture [53].

While there is now a large literature on management in the healthcare sector, very few studies have so far investigated the role of leadership style for physicians. To the best of our knowledge, the only exception from the extensive volume of research focusing on nursing leadership is a contribution from 2014, which investigated whether doctors’ perception of leadership style and organisational culture influenced their organisational commitment [54]. The study used the Multifactor Leadership Questionnaire developed by Avolio and Bass [55] to identify the doctors’ perceptions of leadership behaviour and Hofstede’s organisational culture scale [56] to measure culture. The study found that doctors’ perceptions of leadership behaviour had a statistically significant, positive effect on their level of organisational commitment, and that organisational culture did not act as a moderator in this relationship.

There is thus a strong need for studies of the relationship between leadership style and organisational culture seen from the perspective of physicians, particularly since the changing role of leadership in hospitals can be expected to have affected their working situation. Health systems across the world have implemented reforms that call for a reconsideration of the role of management of hospitals, which is increasingly seen as important for performance. These reforms often fall under the umbrella of New Public Management (NPM), in which the basic idea is to expose public providers to an environment that has similarities to a private market. A central recommendation is to replace traditional public administration with the management principles found in private businesses. There is an emphasis on a clearer manager role: managers should be given the autonomy to lead, but in combination with specific demands for results and goal achievement. In practice, this belief in management implies extensive use of delegation, a professionalisation of the manager role, and that appointments are linked to performance requirements [57]. These reorganisation efforts of the hospitals have challenged and supplemented traditional profession-based management with more complex systems of management inspired by the business sector. In addition, the reforms have introduced competition, patient rights and patient choice, activity-based financing, purchaser-provider models, performance measurements and performance-based contracts. Leaders at all levels of the hospital organisation have therefore been given more autonomy to make key strategic, financial and clinical decisions, which have in turn challenged the role, identity and autonomy of medical professionals [11]. The reforms generally ask clinicians to adopt a perspective that balances clinical autonomy with managerial accountability and to recognise the interconnectedness between the clinical and financial dimensions of care [58]. Several studies have focused on the emerging hybrid medical profession, and how they respond to the new demands of corporate and managed care [58–65]. While there is now much evidence on how medical professionals in their role as leaders and managers adjust to the new institutional logics of the health care sector, we do not know of any previous studies that have investigated whether leaders’ emphasis on clinical or financial priorities is related to how hospital physicians’ view their work situation.

The purpose of this study was therefore to examine the relationship between leadership style and organisational culture as perceived by the hospital physicians. Numerous definitions and ways of measurements of organisational culture exist, but here we employ the more specific concept of organisational climate, which can be viewed as an integrated part of culture [66–68]. The term organisational climate relates to an employee’s psychological attachment to the organisation [69]. Two types of leadership styles were contrasted in the analysis: a traditional profession-based leadership logic that emphasises the promotion of professional standards and quality in patient treatment, and a leadership style that reflects the emerging management philosophy with focus on economic administration and budgetary control. The analysis was performed within the context of the Norwegian hospital sector, where a major NPM-inspired reform was implemented in 2002 (for more information on the reform, see e.g. [70–72]). The reform introduced more autonomy and market-like management practices, thereby aiming to change management conditions. It also introduced corporate principles of accounting not previously used in the public sector, i.e. the introduction of capital costs in hospital budgets and accounts. A main motivation was to give leaders more competence and autonomy by professionalising management and allowing a higher degree of freedom from the political sphere [73].

The same data as employed here was previously used to study hospital physicians’ intention to leave their current job [11]. This study found that a professional-supportive leadership style may have a positive influence on retention of physicians in public hospitals. The relationship between leadership style and doctors’ organisational climate is of particular interest in a hospital setting: whereas external conditions such as institutional and environmental aspects are difficult to influence, leadership strategies and styles are something that can be changed within the organisation itself. After all, leadership strategies can be developed through various methods, such as workshops, training sessions or self-improvement methods [74]. Therefore, identifying leader-specific predictors of organisational climate could lead to development of special recruitment and retention strategies for hospitals with high risk of a poor organisational climate.

## Methods

We utilised data from a survey among 3000 Norwegian hospital physicians from 2016, undertaken in collaboration with The Norwegian Medical Association (NMA). The respondents were recruited via sampling from the NMA register of members fully employed in a public hospital. Of the gross sample of 2967 respondents, 971 ended up answering the survey, which gives a response rate of 33 %. Whereas this may cause some concern, it is still uncertain whether a low response rate necessarily results in skewed samples and lower representativeness [75, 76]. Furthermore, we were able to assess the representativeness of our sample by comparing it with the members of the register of the NMA and found that the respondents in our data deviated marginally (only between 0 and 3 %) from the register. All methods were performed in accordance with the relevant guidelines and regulations.

### Dependent variables

The analysis utilised three additive indexes as dependent variables to reflect various aspects of the organisational climate: *social climate*, *innovation climate* and *engagement* at the workplace. The questions used to construct the variables were taken from an internationally recognised scale on organisational climate; The General Nordic questionnaire for psychological and social factors at work (QPSNordic). QPSNordic has been thoroughly tested and tried in many organisations, and is used, among others, by the National Institute of Occupational Health in Norway [77]. Correlation and reliability tests showed that the items were suitable for additive indexes in all three cases (results not shown here).

#### Social climate

Social climate was measured through an additive index based on the respondents’ rankings of the social climate in their work unit on the following three statements, with 5-point Likert scales as response format (1 = ‘very little/not at all’, 5 = ‘very much’): *“encouraging and empowering”*, *“relaxed and agreeable”* and *“rigid and rule-oriented”* (Table 1). The direction of the last variable was reversed for the analysis.

**Table 1:** “How is the social climate in your work unit”? Per cent with frequencies in parenthesis

	1 = “Very little/not at all”	2 = “Quite little”	3 = “Some”	4 = “Quite much”	5 = “Very much”	N
“Encouraging and empower-ing”	7.4 (70)	15.9 (151)	29.2 (278)	37.4 (356)	10.1 (96)	951
“Relaxed and agreeable”	7.7 (73)	18.8 (177)	33.2 (313)	31.8 (300)	8.5 (80)	943
“Rigid and rule-oriented”	10.4 (99)	37.3 (354)	27.2 (258)	18.6 (176)	6.4 (61)	948

#### Innovation climate

Innovation climate was measured through an additive index based on the following three questions about the innovation climate in the work unit, with 5-point Likert scales as response format (1 = ‘very rarely or never’, 5 = ‘very often or always’): *“Do the employees take initiatives themselves at your workplace?”*, *“Are employees encouraged to think of ways of doing things better at your workplace?”* and *“Is the communication good enough in your department?”* (Table 2).

**Table 2:** “How is the innovation climate in your work unit”? Per cent with frequencies in parenthesis

	1 = “Very rarely or never”	2 = “Quite rarely”	3 = “Sometimes”	4 = “Quite often”	5 = “Very often or always”	N
“Do the employees take initiatives themselves at the workplace?”	3.7 (35)	13.6 (129)	34.3 (326)	41.3 (393)	7.2 (68)	951
“Are employees encouraged to think of ways of doing things better in the workplace?”	10.8 (103)	20.5 (195)	33.9 (322)	27.7 (263)	7.1 (67)	950
“Is the communication good enough in your department?”	7.3 (69)	18.4 (174)	28.5 (269)	36.1 (341)	9.6 (91)	944

#### Engagement

The engagement at the workplace was measured through an additive index based on the respondents’ assessments of the engagement in their work unit on the following three statements, with 5-point Likert scales as response format (1 = ‘strongly disagree’, 5 = ‘strongly agree’): *“I tell my friends that this is a good organisation to work in”*, *“My values are very much alike the values of the organisation”*, and *“This organisation really inspires me to do my best”* (Table 3).

**Table 3:** “How is the engagement in your work unit”? Per cent with frequencies in parenthesis

	1 = “Fully disagree”	2 = “Partly disagree”	3 = “Neither agree or disagree”	4 = “Partly agree”	5 = “Fully agree”	N
“I tell my friends that this is a good organisation to work in”	11.7 (111)	19.5 (185)	17.2 (164)	31.4 (299)	20.2 (192)	951
“My values are very much alike the values of the organisation”	12.3 (117)	28.5 (271)	23.4 (222)	26.2 (249)	9.6 (91)	950
“This organisation really inspires me to do my best”	17.4 (165)	26.6 (253)	21.8 (207)	25.4 (241)	8.8 (84)	950

### Leadership style

The central independent variables in the analysis are leadership style. These were based on an item in the survey asking the respondents to rate the leadership qualities of their proximate leaders (department chair) on the following 11 specific dimensions on a 5-point Likert scale (1 = “does not emphasise”, 5 = “emphasises very much”):

- ‘Ensure professional standards and quality in patient treatment’
- ‘Stimulate professional collaboration across different departments’
- ‘Motivate employees and create support’
- ‘Develop and utilize new routines and working methods’
- ‘Coordinate different types of activity within the department’
- ‘Solve interpersonal problems and differences’
- ‘Initialize new professional opportunities’
- ‘Economic steering, accounting and budget’
- ‘Ensure that rules and routines are followed’

We first used factor analysis to identify different types of leadership styles. The factor analysis showed that the variables loaded on two factors (for more details on the factor analysis, see [11]): one dimension with focus on professional and motivational aspects (items a-g), and one dimension with emphasis on economic and administrative aspects (items h and i). Based on this, we constructed two different additive variables for leadership style, where one reflects what we have labelled a *professional-supportive style*, while the other reflects an *economic-operational style*.

### Controls

We also included several control variables to take into account aspects of the hospital physicians’ background that may be relevant for how they assess the organisational climate. The control variables were selected on the basis of previous research and similar studies [11, 20, 78–82]. First, we included the respondents’ *demographic* background, captured through age and gender. Second, the model also incorporated several variables to reflect the physicians’ professional background. A dummy-variable captured whether the respondent holds a *leader* role or not. Furthermore, a considerable share of physicians working in Norway have a medical exam from abroad, and thus have their training from an organisational and institutional setting that may differ from a Norwegian hospital context. Thus, *foreign medical exam* was also included as a possible confounding factor. We also controlled for *specialty* by entering dummy-variables for internal medicine, laboratory, psychiatry and “other”, with surgery as the reference category.

Finally, there is a possibility that a strong scepticism among the hospital physicians towards the general hospital organisation model might affect their evaluations of their leaders. It has been documented that the hospital physicians are generally negative towards the enterprise model that was introduced with the hospital reform of 2002 [11], and in order to account for such possible confounding effects, we included a variable reflecting the respondents’ view on the enterprise model.

Given that the data set at hand also included information on the respondents’ hospital affiliation, the data set is hierarchically structured, with physicians nested in hospitals. We should therefore consider multi-level modelling as methodological approach, but initial tests showed that less than 5 % of the variance in the dependent variables could be ascribed to the hospital-level. Hence, there were no statistical reasons to continue with multi-level modelling, and the models were estimated with OLS regression.

The descriptive statistics are presented in Table 4. Since several of the independent variables in our model may be highly correlated, there is a potential concern for imprecise estimates due to large variance. However, collinearity diagnostics uncovered no such problems.

**Table 4:** Descriptive statistics

Variables		N
Professional-supportive leadership style	Mean: 2.87 Min.: 1 Max.: 5 St. dev.: 0.85	912
Economic-operational leadership style	Mean: 3.94 Min.: 1 Max.: 5 St. dev.: 0.65	932
Leadership responsibilities (yes = 1, no = 0)	0: 698 (80.0 %) 1: 190 (20.0 %)	953
Age (< 40 = 1, > 40 = 0)	0: 760 (73.2 %) 1: 255 (26.8 %)	950
Gender (female = 1, male = 0)	0: 567 (59.6 %) 1: 385 (40.4 %)	952
Surgery	0: 589 (61.5 %) 1: 368 (38.5 %)	957
Internal medicine	0: 627 (65.5 %) 1: 330 (34.5 %)	957
Laboratory	0: 865 (90.4 %) 1: 92 (9.6 %)	957
Psychiatry	0: 823 (86.0 %) 1: 134 (14.0 %)	957
Other	0: 925 (96.7 %) 1: 32 (3.3 %)	957
Critical towards the enterprise model (yes = 1, no = 0)	0: 503 (53.5 %) 1: 437 (46.5 %)	940
Social climate index	Mean: 3.23 Min.: 1 Max.: 5 St. dev.: 0.91	940
Innovation climate index	Mean: 3.19 Min.: 1 Max.: 5 St. dev.: 0.88	941
Engagement index	Mean: 3.01 Min.: 1 Max.: 5 St. dev.: 1.14	947

## Results

Table 5 shows the results for the regression on the three dependent variables representing organisational climate. As can be seen, a professional-supportive leadership style is positively associated with how the social climate is perceived (*B* = 0.51, *p <* .00), while the opposite is the case for economic-operational leaderships (*B* = − 0.31, *p* < .00). Turning to the second indicator of organisational climate, the innovation climate, we found the same relationship for professional-supportive leadership: the more the physicians report that their closest leaders emphasise professional-supportive aspects, the better they perceive the innovation climate (*B* = 0.63, *p* < .00). However, the results uncover no significant association between the economic-operational style and view on innovation climate. We also observe the same tendency for the last organisational climate variable: engagement is positively related to professional-supportive leadership (*B = .*81, *p* < .00), while economic-operational leadership is without significance.

**Table 5:** Relationship between department leadership style and organisational climate. Estimated via OLS regression. Standardised Beta coefficients with standard errors in parenthesis

	(1) Social climate	(2) Innovation climate	(3) Engagement
*Leadership style*:			
Professional-supportive leadership	0.562** (0.031)	0.633** (0.029)	0.811** (0.036)
Economic-operational leadership	− 0.056* (0.039)	0.021 (0.036)	0.030 (0.045)
*Controls*:			
Leader responsibility (yes = 1, no = 0)	0.034 (0.061)	0.141* (0.057)	0.175* (0.071)
Age (< 40 = 1, > 40 = 0)	0.014 (0.065)	0.018 (0.061)	0.031 (0.076)
Gender (female = 1, male = 0)	− 0.028 (0.053)	− 0.056 (0.050)	0.037 (0.062)
Foreign medical degree (yes = 1, no = 0)	0.058* (0.054)	0.042 (0.050)	0.059 (0.063)
Internal medicine	− 0.063 (0.059)	− 0.028 (0.055)	0.038 (0.069)
Laboratory	− 0.068 (0.092)	− 0.033 (0.086)	− 0.063 (0.107)
Psychiatry	0.042 (0.078)	0.031 (0.074)	0.159 (0.091)
Other	0.002 (0.155)	− 0.073 (0.145)	0.317 (0.181)
Critical towards the enterprise model (yes = 1, no = 0)	− 0.072* (0.053)	− 0.043 (0.048)	− 0.320** (0.061)
Intercept	1.821** (0.182)	1.288** (0.049)	0.581** (0.212)
N	853	854	856
R2 adjusted	0.353	0.400	0.443

** *p* < .01, * *p* < .05

Turning to the control variables, it is worth noticing that respondents who hold a leader position as expected rate both the innovation climate and the engagement more favourably than those without such responsibility. Similarly, respondents who are critical towards the enterprise model tend to rate both the social climate and engagement as less favourable.

## Discussion

A central aspect of many recent health reforms has been the introduction of management principles and quasi markets. The reforms have typically been motivated by a (perceived) need to improve equity, quality and efficiency. However, a common concern is that they may increase the emphasis on economic aspects of health care, thereby challenging the medical principles that guide hospital activities. This study investigated the relationship between leadership style and organisational climate in hospitals as viewed by the physicians. Does the emphasis and priorities of clinical leaders matter for the organisational climate, or are they so bound by structural boundaries that there is little room for exercising leadership? The results here document a clear relationship between leadership style and how organisational climate is assessed: a leadership style which is perceived to promote professional standards and quality in patient treatment is associated with a better evaluation of both social climate, innovation climate and engagement at the workplace, while an emphasis on economic management and budgetary control is associated with a poorer social climate.

As with similar health reforms in other countries, the Norwegian reform introduced new incentives, ideas and management principles previously unknown to hospital governance, allowing for new leadership practices inspired by the private sector. A new generation of managers and executives have thus entered the hospitals, embodying the philosophy of NPM to ‘let leaders lead’, and embracing the central keywords typically associated with the NPM doctrine: distinct objectives, output demands, and, not least, skilled and genuine leadership. In times of tight healthcare budgets and a general increasing demand for health services, managers and clinical leaders are typically given more autonomy and freedom from bureaucracy and politicians. This context allows for new and more important roles of leadership, where clinical leaders are able to exert more influence than before.

This introduction of private sector style management, quasi-markets, purchaser-provider models and performance indicators into healthcare has stimulated a debate on ‘hybridisation’ of managerial and professional approaches. The hybridisation thesis argues that medical professionals have willingly adopted managerial, financial and accounting discourses, thus leading to the hybridisation of medical expertise, framed by both professionalism and managerial logics [83–88]. Alternatively, the polarisation thesis suggests that medical professionals resist the culture of managerialism because it fundamentally clashes with the culture of professional autonomy and clinical values. Instead, a separate subgroup is expected to emerge to manage financial and administrative responsibilities, leaving the fundamental values and practices of the wider profession unchanged [89–93]. In the case of Norway, earlier research suggests that there is heterogeneity within the profession rather than managerialist values colonising the medical profession through a process of hybridisation: some physician managers adopt management values and tools, whereas others remain alienated from them [94]. The results reported here seems to support such a view, given that economic-operational management is negatively associated with the social climate.

The concept of organisational culture has been variably and extensively defined, measured and researched for several decades. The interest of organisational behaviour researchers in the concept stems from the belief that culture is important for an organisation’s performance. This study focused on the concept of organisational climate, which is the meanings people attach to interrelated bundles of experiences they have at work [67]. The research interest has been directed both towards the theoretical development of constructs and measurements and towards empirical efforts to uncover the antecedents and outcomes of organisational climate (for overviews, see e.g. [95, 96]). A considerable literature has looked specifically at the relationship between leadership and organisational climate [97–103]. The contribution of this study is to investigate the relationship between leadership style and organisational climate as viewed by hospital physicians. To the best of our knowledge, this is the first study to do so. Physicians is a professional group that represent a particularly interesting case in the context of organisational climate.

Furthermore, there is now a large volume of studies documenting the positive impact of employee-oriented and supportive leadership on safety, quality, clinical performance, service improvement and other important outcomes, as well as on various aspects of organisational culture. Given that our operationalisation of the professional-supportive leadership style reflects some of the central qualities of transactional leadership, we should not be surprised that this mode of leadership is positively viewed also by hospital physicians. The other main contribution of this study is that it investigated the role of professional leadership as well, in addition to supportive leadership. As far as we know, this has not been done before. In the case of physicians, the professional dimension of leadership is likely to become increasingly important in the wake of the many NPM-inspired reforms in healthcare. The medical professionals belong to a self-regulating professional community, within which socialisation and internalisation of common norms takes place through education and collective discipline based on specialised knowledge. This mode of regulation is often referred to as ‘medicratic’ regulation – as opposed to public hierarchies and markets [104]. Medical professionals thus bear much resemblance with the term ‘cosmopolitans’, which was introduced by Gouldner [105]: being low on loyalty to the employing organisation, high on commitment to specialised role skills, and likely to use an outer reference group orientation. In the same way, the medical professionals’ frame of reference could be expected to be first and foremost directed towards their profession rather than towards the hospital organisation itself, as they seek recognition and acceptance from their peers rather than from outsiders. Consequently, for physicians there is a strong relationship between perceiving to be led in a professional way and how they assess the organisational climate.

There are some limitations with our study that should be addressed. Most importantly, our analyses are unable to say anything about causality. Given that the study used cross section data we are only able to assess covariation and cannot exclude the possibility that causality works the opposite direction; i.e. that those who report a favourable organisational climate also have a higher tendency to assess their leaders as prioritising professional and supportive aspects. In order to assess causality, we would need information on changes over time.

Also, it could be maintained that the traits associated with the economic-operational leadership style would be difficult for anyone to perceive as supporting social, innovative and engagement climate in an organisation. Rather, it might be the opposite, since our definition of economic-operational leadership is narrowed down to two questions: “economic steering, accounting and budget” and “ensure that rules and routines are followed”. Hence, it could be argued that all the positive traits are associated with professional-supportive leadership and the negative traits (in matter of organisational climate) to economical-operational leadership. Therefore, in order to isolate the effects of professional leadership we decomposed the professional-supportive leadership style into two separate variables for *professiona*l traits (“ensure professional standards and quality in patient treatment”, “stimulate professional collaboration across different departments” and “initialise new professional opportunities”) and *supportive* traits (“motivate employees and create support” and “solve interpersonal problems and differences”). The new analyses showed that these two dimensions are both significantly associated with all three aspects of organisational climate, and that the magnitude of their relevance is about the same (results not shown here).

Another limitation is that there are several relevant explanatory factors of organisational climate that we were unable to control for in our model due to the lack of such information in the data. The most obvious variables include salary, working hours and patient load. Other potentially relevant control variables could be whether department leaders have any formal management education and their experience as leaders. Also, whereas the initial tests showed that very little of the variation in organisational climate can be ascribed to the hospital level, it would have been interesting to investigate possible variation that could be ascribed to the department level. If such data were available, we could have controlled for relevant department-specific characteristics such as size, patient-mix, economy, etc.

Furthermore, it may unclear how close the physicians are to the leaders they report on. Whereas the question in the survey asked about their “proximate leader”, we cannot rule out that this could sometimes be leaders at the next level and further away from the respondents. Since the survey also asked the respondents to rate the qualities of their leaders at the enterprise level, we therefore tried estimating the same model to uncover the relevance of leadership style at this level. The results from the analyses are not reported here, but they were about the same as for the analyses of “proximate leaders”.

Finally, while our data was collected in 2016, we would maintain that they are still very relevant. The resistance to NPM ideas in general, and to the enterprise model in particular, has always been strong in the Norwegian medical profession, and there is little reason to expect it to have lessened. If anything, the opposition towards the “enterprise logic” has probably only increased since the data was collected [106–110]. It is therefore reasonable to assume that more recent data would only strengthen the results documented here.

## Conclusions

We know little about how leadership style is related to hospital physicians’ view of the organisational culture. This study suggests that leadership indeed matters: a leadership style that emphasises professional traits – in addition to being supportive – is associated with better social climate, innovation climate and engagement in hospitals, while an economic-operational leadership style is related to a poorer social climate. The cross-sectional study design makes it impossible to draw inferences about direction of causality and causal pathways. However, the positive relationship between professional-supportive leadership and organisational climate is a matter, which should be seriously considered regardless of direction of causality.

## Data Availability

The data sets used and/or analysed during the current study are available from the corresponding author on reasonable request.
